# Human Sulfotransferase Assays With PAPS Production *in situ*

**DOI:** 10.3389/fmolb.2022.827638

**Published:** 2022-02-28

**Authors:** Yanan Sun, Lukas Corbinian Harps, Matthias Bureik, Maria Kristina Parr

**Affiliations:** ^1^ Pharmaceutical and Medicinal Chemistry (Pharmaceutical Analyses), Institute of Pharmacy, Freie Universitaet Berlin, Berlin, Germany; ^2^ School of Pharmaceutical Science and Technology, Health Sciences Platform, Tianjin University, Tianjin, China

**Keywords:** fission yeast, *in vitro* metabolism, method optimization, PAPS, sulfonation, SULT, quality by design, isotopic labelling

## Abstract

For *in vitro* investigations on human sulfotransferase (SULT) catalyzed phase II metabolism, the costly cofactor 3′-phosphoadenosine-5′-phosphosulfate (PAPS) is generally needed. In the present study, we developed and optimized a new approach that combines SULT-dependent biotransformation using recombinant and permeabilized fission yeast cells (enzyme bags) with PAPS production *in situ* applying quality by design principles. In the initial application of the procedure, yeast cells expressing human SULT1A3 were used for the production of 4′-hydroxypropranolol-4-*O*-sulfate from 4-hydroxypropranolol. The optimized protocol was then successfully transferred to other sulfonation reactions catalyzed by SULT2A1, SULT1E1, or SULT1B1. The concomitant degradation of some sulfoconjugates was investigated, and further optimization of the reaction conditions was performed in order to reduce product loss. Also, the production of stable isotope labelled sulfoconjugates was demonstrated utilizing isotopically labelled substrates or ^34^S-sulfate. Overall, this new approach results in higher space-time yields while at the same time reducing experimental cost.

## Introduction

The study of metabolic pathways of drug substances in humans relies both on *in vivo* and *in vitro* experiments. After administration drugs are either directly excreted unchanged or metabolized first. The main specimen for excretion of most drugs and metabolites is urine. The parent drugs often show shorter detection windows due to extensive metabolism. Therefore, in toxicological, forensic or doping control analysis metabolites are often used as target analytes in urine samples (Balcells et al., 2017; Esquivel et al., 2019). Even though *in vivo* techniques are widely applied in anti-doping research, the high expenditure and unpredicted toxicological effect of many prohibited drugs are considerable drawbacks. In the last two decades, modern *in vitro* techniques became a viable alternative and furthermore a great extension to *in vivo* studies (Ekins et al., 2000). Moreover, they allow for precise reaction phenotyping. In *in vitro* studies, tissues or fractions of tissues like liver microsomes or homogenized liver fractions are commonly applied. In recent years, biosynthesis of sulfoconjugates using genetically modified microorganisms has been developed as well (Taskinen et al., 2003; Nishikawa et al., 2018). Metabolism of drug substances occurs in the complementary phases I and II. While enzymes of phase I metabolism transform parent compounds by hydroxylation, oxidation, or reduction, phase II metabolism consists of the attachment of small moieties to the target molecules, thus allowing them to be excreted from the body rapidly and efficiently. The majority of phase II metabolites are glucuronide- or sulfoconjugates. The formation of the latter species is catalyzed by sulfotransferases (SULTs), which for their activity depend on the cofactor 3′-phosphoadenosine-5′-phosphosulfate (PAPS).

For laboratory use PAPS is highly expensive (approx. 286 US$/mg). This fact might contribute to the limited number of sulfonation studies as compared with research on glucuronidation. In biological sulfonation, conversion of inorganic sulfate into the high-energy cofactor PAPS is a prerequisite. In this pathway, adenosine triphosphate (ATP) sulfonation is initially catalyzed by ATP-sulfurylase to generate adenosine-5′-phosphosulfate (APS), which is subsequently phosphorylated by APS kinase to yield PAPS (Burkart et al., 2000). Afterwards, the sulfo-group is transferred from PAPS to the parent drug or its phase I metabolite in a reaction catalyzed by a SULT enzyme. The released 3′‐phosphoadenosine‐5′‐phosphate (PAP) is subsequently dephosphorylated and re‐phosphorylated in several enzymatically catalyzed steps to regenerate ATP (Robbins and Lipmann, 1958). In animal cells, ATP sulfurylase and APS kinase are expressed as a bifunctional enzyme named PAPS synthase (PAPSS), whereas in bacteria, yeasts, fungi, and plants, the two enzymes are generally encoded by separate genes (Besset et al., 2000).

In the budding yeast *Saccharomyces cerevisiae* ATP sulfurylase and APS kinase are encoded by the genes MET3 and MET14, respectively (Masselot and Surdin-Kerjan, 1977; Cherest et al., 1985; Cherest et al., 1987; Mountain and Korch, 1991). In the fission yeast *Schizosaccharomyces pombe* there is a MET14 homologue which is predicted to encode a APS kinase (Lock et al., 2019). While this has yet to be demonstrated experimentally, PAPS synthesis in *S. pombe* as such has already been reported (Song and Roe, 2008). Previously, all 14 human SULTs have been functionally expressed in *S. pombe* and, moreover, using this microbial host the functionality of SULT4A1 and SULT6B1 was demonstrated for the first time (Sun et al., 2020). In comparison to whole-cell biotransformation, sulfonation of drugs with permeabilized recombinant fission yeast cells (enzyme bags) provided higher sensitivity and shorter reaction times. However, the required PAPS addition makes this approach expensive for extensive substrate screening or for up-scaling of biosynthetic metabolite production. In this study, we investigated the possibility of generating PAPS by endogenous fission yeast enzymes in the presence of (comparatively cheaper) ATP and ammonium sulfate. For this purpose, the experimental conditions were first optimized using SULT1A3 and 4-hydroxypropranolol (4HP) as model compound. Further verification was then performed with several other SULTs and substrates.

## Material and Methods

### Chemicals and Reagents

Na_2_HPO_4_ and CaCl_2_ • 2 H_2_O were purchased from Riedel de Haen (Seelze, Germany). KH_2_PO_4_, CuSO_4_ • 5 H_2_O, H_3_BO_3_, potassium hydrogen phthalate, Na_2_SO_4_, nicotinic acid, MnSO_4_ • H_2_O, and KI were from Merck (Darmstadt, Germany). ZnSO_4_ • 7 H_2_O was purchased from Acros (Geel, Belgium). Tris, agar, NH_4_HCO_3_, NH_4_Cl, FeCl_3_ • 6 H_2_O, MgCl_2_ • 6 H_2_O, glucose, Triton-X100, and biotin were from Roth (Karlsruhe, Germany). Inositol was from Th.Geyer (Berlin, Germany), and MoO_4_ • 2 H_2_O was purchased from Alfa Aesar (Kandel, Germany). Dehydroepiandrosterone (DHEA) was obtained from Steraloids (Newport, RI, United States). 4HP, ATP, and citric acid were purchased from Sigma Aldrich (Steinheim, Germany). 7-Hydroxycoumarin (7HC) and formic acid were purchased from TCI (Zwijindrecht, Belgium). ^34^S labelled (NH_4_)_2_SO_4_ and D_9_-Salbutamol (D_9_-SA) were purchased from Sigma-Aldrich (Taufkirchen, Germany), D_6_-DHEA was obtained from Sigma-Aldrich (Saint Louis, MO, United States). Acetonitrile was from Fischer Scientific (Geel, Belgium), and HCOONH_4_ was from VWR Chemicals (Damstadt, Germany). Ultrapure water was prepared with a Milli-Q water purification system LaboStar 2-DI/UV from SG Wasseraufbereitung und Regenerierstation GmbH (Barsbüttel, Germany). All other chemicals and reagents used were also of the highest grade available.

### Fission Yeast Strains, Media and General Techniques

The recombined fission yeast strains YN3, YN4, YN20, and YN25 used in this project were described before (Sun et al., 2020). The preparation of media and basic manipulation methods of *S. pombe* were carried out as described (Alfa et al., 1993). Briefly, strains were generally cultivated at 30°C in Edinburgh Minimal Medium (EMM). EMM was prepared with NH_4_Cl (93.5 mM), glucose (2% w/v), Na_2_HPO_4_ (15.5 mM), potassium hydrogen phthalate (14.7 mM) and standard amounts of salt, vitamin and mineral stock solutions. Liquid cultures were kept shaking at 230 rpm.

### Biotransformation With Enzyme Bags

This was essentially done as described before (Sun et al., 2020) with slight modifications. Briefly, fission yeast strains were grown in 10 ml liquid culture of EMM at 30°C and 230 rpm for 24 h. Incubation of main cultures in 250 ml Erlenmeyer flasks was performed subsequently. For each assay a certain number of cells were transferred to micro centrifuge tubes or falcons, pelleted and incubated in 0.3% Triton-X100 in Tris-KCl buffer (200 mM KCl, 100 mM Tris-Cl pH 7.8) at 30°C for 60 min at 230 rpm to allow for permeabilization. Cells were then washed thrice with NH_4_HCO_3_ buffer (50 mM, pH 7.8) and directly used for SULT-dependent reactions. Enzyme bags were resuspended in 200 µL of aqueous NH_4_HCO_3_ buffer (50 mM, pH 7.8) or phosphate buffer (50 mM, pH 7.8) containing PAPS or ATP, ammonium sulfate, magnesium chloride and substrate as indicated. Biotransformations were carried out at 37°C in a shaking incubator (300 rpm). Enzymatic reactions were stopped by short sharp centrifugation at 14,100 rcf for 2 min and 200 µL of sample in 1.5 ml micro centrifuge tubes were directly frozen at –20°C. After defrosting, samples were centrifuged again (14,100 rcf, 2 min). Supernatants were directly analyzed by ultra-high performance liquid chromatography tandem mass spectrometry (UHPLC-MS/MS) or diluted with a mixture of acetonitrile and ultrapure water (50/50, v/v) prior to analysis. Negative control samples were incubated without cofactors (ATP, (NH_4_)_2_SO_4_, and MgCl_2_) or without cells, respectively.

### Multifactorial Optimization

The optimization process was performed applying quality-by-design (QbD) principles. Design of experiments (DoE) was used for multivariate statistical analysis aiming to ensure robust protocol conditions. It started with a broad systematic screening of the influence of several factors on the incubation of 4HP with YN20 (SULT1A3): ATP concentration (1–50 mM, while (NH_4_)_2_SO_4_ concentration was always kept to the half of ATP concentration), incubation time (3–72 h), cell number per incubation, pre-incubation time, and magnesium chloride concentration (1–100 mM) were altered. Results of the pre-screening disclosed some limits and trends of the factors. For further optimization a Box-Behnken design was used to investigate the effect of the five dependent variables in biotransformation and to optimize the experimental conditions to achieve the highest yield. The variables in this design involved ATP concentration (11–20 mM), magnesium chloride concentration (10–100 mM), pre-incubation time (i.e. incubation without substrate for either 0, 3, or 5 h), incubation time (3–24 h), and cell number (either 5 $\times 10^{7}$ , 1.25 $\times 10^{8}$ , 2.5 $\times 10^{8}$ , or 5 $\times 10^{8}\text{ per }200\text{ µl}$ ). Considering the outcome and prediction of pre-screening and first round of optimization two further rounds of fine tuning were performed subsequently. The best conditions of round two and three as well as predicted optimal conditions were then compared as proof of concept and re-evaluated focusing on incubation time in particular. Also, both NH_4_HCO_3_ and phosphate buffer systems were evaluated. Product formation was monitored by UHPLC-MS/MS and results of the same analytes were compared *via* peak area. Statistical data analysis and parts of experimental design were carried out using Minitab (RRID:SCR_014483, Statistical Software, Coventry, United Kingdom) software program.

### Biosynthesis of Isotope-Labelled Metabolites and Evaluation of *in situ* PAPS Generation

D_6_-DHEA (100 µM) and D_9_-SA (100 µM) were used as substrates in enzyme bags experiments. Enzyme bags experiments were also carried out with DHEA (100 µM) and SA (100 µM) utilizing (NH_4_)_2_^34^SO_4_ (5.5 mM) and ATP (11 mM) as educts for the cofactor PAPS. Production of labelled sulfoconjugates was monitored by UHPLC-MS/MS.

### Degradation Experiments

Degradation of an already sulfonated metabolite in enzyme bags experiment was tested by incubating 7-hydroxycoumarin sulfate (7HCSU) with either YN3 or YN4 for 5 h at 37°C. All experiments were carried out in duplicates.

### Optimized Enzyme Bags Biosynthesis

Based on the optimization experiments the final enzyme bag method used 2.5 × 10^8^ precultured and pelleted fission yeast cells that are permeabilized using 200 µL of Triton-X100 [0.3% in Tris-KCl buffer (200 mM KCl, 100 mM Tris-Cl pH 7.8)] at 30°C for 60 min at 230 rpm. After washing with NH_4_HCO_3_ buffer (50 mM, pH 7.8, three times) enzyme bags were resuspended in 200 µL of aqueous NH_4_HCO_3_ buffer (50 mM, pH 7.8) and supplied with ATP at 11 mM, ammonium sulfate at 22 mM, and magnesium chloride at 20 mM. Following substrate addition (final concentration of 100 µM in incubation solution) mixtures are incubated at 37°C at 300 rpm. Sharp centrifugation at 14,100 rcf for 2 min followed by a freeze-thaw cycle at −20°C and a second centrifugation at 14,100 rcf for 2 min yielded the sulfoconjugates in the supernatant.

### UHPLC-MS/MS Instrumentation and Analytical Methods

Separation was conducted on a 1290 Infinity UHPLC System (Agilent Technologies, Waldbronn, Germany) with an Agilent InfinityLab Poroshell 120 Phenyl Hexyl (100 × 2.1 mm, 2.7 µm) column or an Agilent InfinityLab Poroshell 120 C18-EC (2.1 × 50 mm, 1.9 µm) column. As mass detector an Agilent 6495 Triple Quadrupole MS/MS was utilized. The details of the chromatographic separation conditions, the MS/MS operating parameters and the transitions for all analytes are listed in the supplemental information (Supplementary Tables S1–S3). Presented peak areas were provided by transitions of highest intensity (quantifier).

### Statistical Analysis

All data are presented as mean ± SD. Statistical analysis was done using Origin 2021 (Originlab Corporation, Northampton, MA. United States).

## Results

### Optimization of Reaction Conditions for Sulfoconjugate Production With Enzyme Bags

**FIGURE 1:**
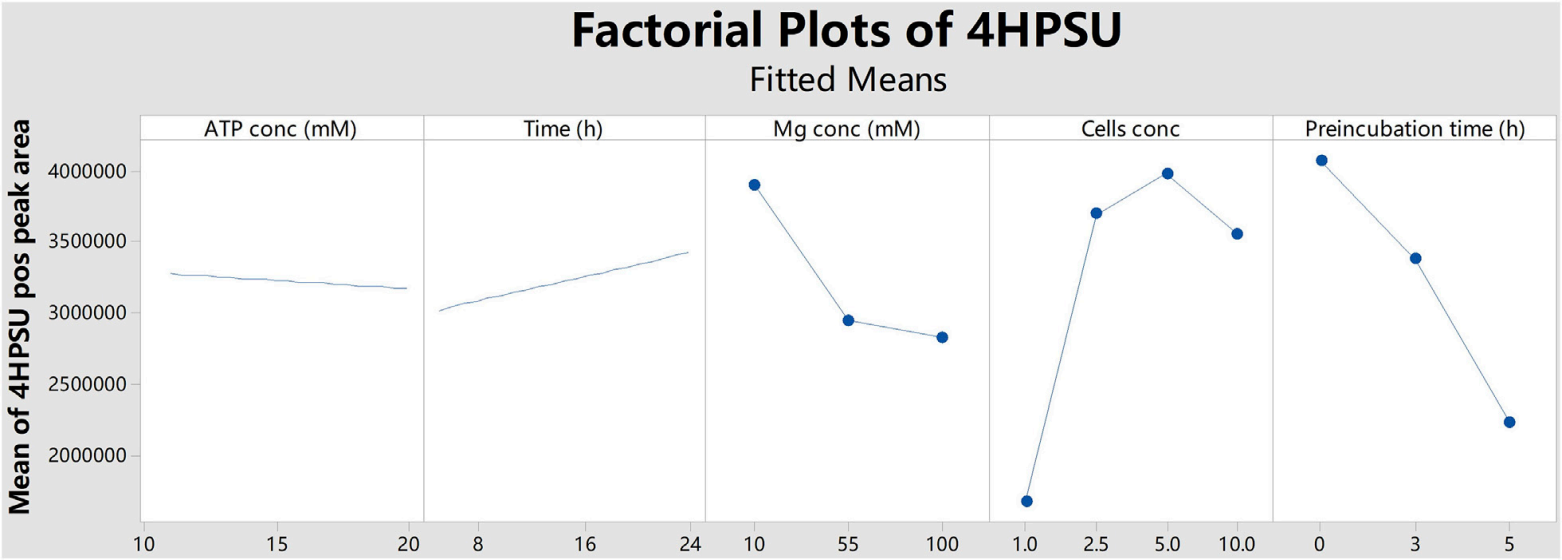
Factorial plots of 4HPSU production with different ATP concentrations (ATP conc), reaction times (Time), MgCl_2_ concentrations (Mg conc), cells concentrations (Cells conc), and preincubation times (Preincubation time). The peak areas of 4HPSU were obtained in positive ion mode and mean values were calculated by Minitab.

**FIGURE 2:**
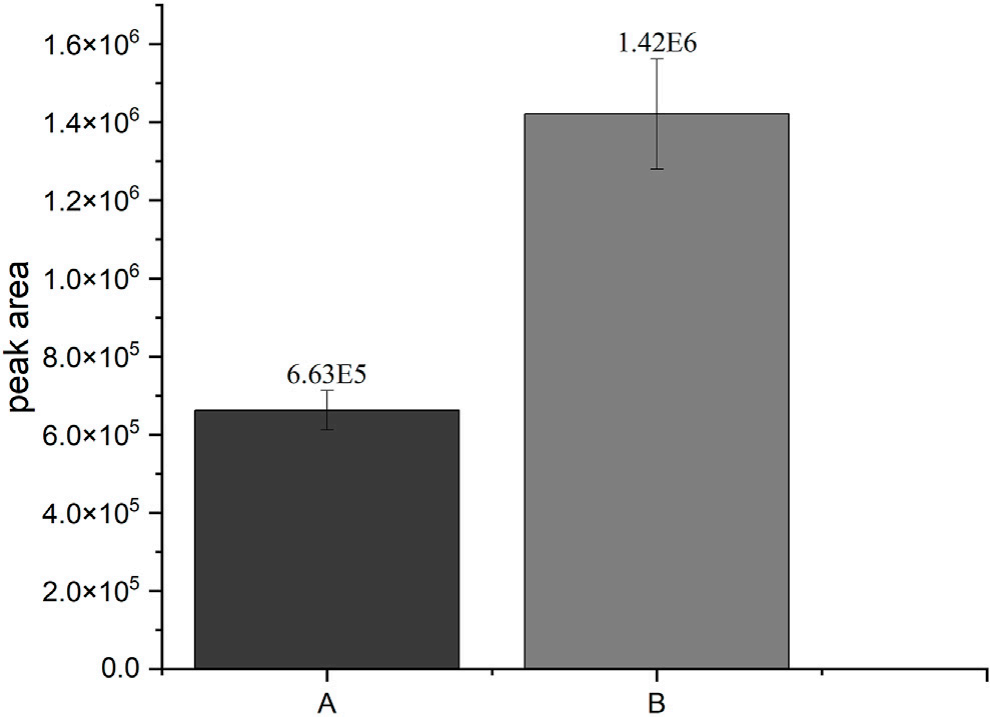
Comparison of the SULT1A3-dependend production of 4HPSU from 4HP under different conditions. A: Peak area of 4HPSU produced with 100 µM PAPS after 3 h incubation; B: Peak area of 4HPSU produced under optimized conditions (11 mM ATP, 20 mM MgCl_2_, 2.5 × 10^8^ cells per sample, and after 5 h incubation time). Each experiment was done in triplicates. Error bars show the standard deviations.

Initially, a sulfonation assay of 4HP (for reaction schemes see Supplementary Figure S1) by SULT1A3 (strain YN20) with external PAPS (100 µM) was carried out as described (Sun et al., 2020). For substitution of the cofactor PAPS by ATP and SO_4_^2-^ and optimization of the product yields multifactorial screening supported by Minitab software was started with five factors: ATP concentration, MgCl_2_ concentration, number of cells, reaction incubation time, and time of preincubation without substrate. Initial results indicated that a reduction of yield was correlated with longer preincubation time (Figure 1). Therefore, later rounds were performed without any preincubation. Two optimization rounds were conducted within a more targeted range of each factor using Box-Behnken design. More specifically, ATP concentrations of 11 mM or 15 mM, cell numbers of 1.25 $\times 10^{8}$ , 2.5 $\times 10^{8}$ , or 5 $\times 10^{8}$ per assay, MgCl_2_ concentrations of 10 mM, 20 mM or 30 mM, and incubation times from 5 to 72 h were tested. The optimal conditions obtained were 11 mM ATP, 2.5 $\times 10^{8}$ cells per sample, 20 mM MgCl_2_, and 5 h incubation time. The experiments under the optimal conditions gave over two times higher peak areas of 4-hydroxypropranolol-4-*O*-sulfate (4HPSU) in comparison to the initial reaction with external PAPS (Figure 2). Thus, a standard protocol was established which allows substrate sulfonation with human SULTs recombinantly expressed in fission yeast using ATP and (NH4)_2_SO_4_ instead of PAPS.

### Evaluation of Optimal Conditions for Additional Substrates and Enzyme Isoforms

**FIGURE 3:**
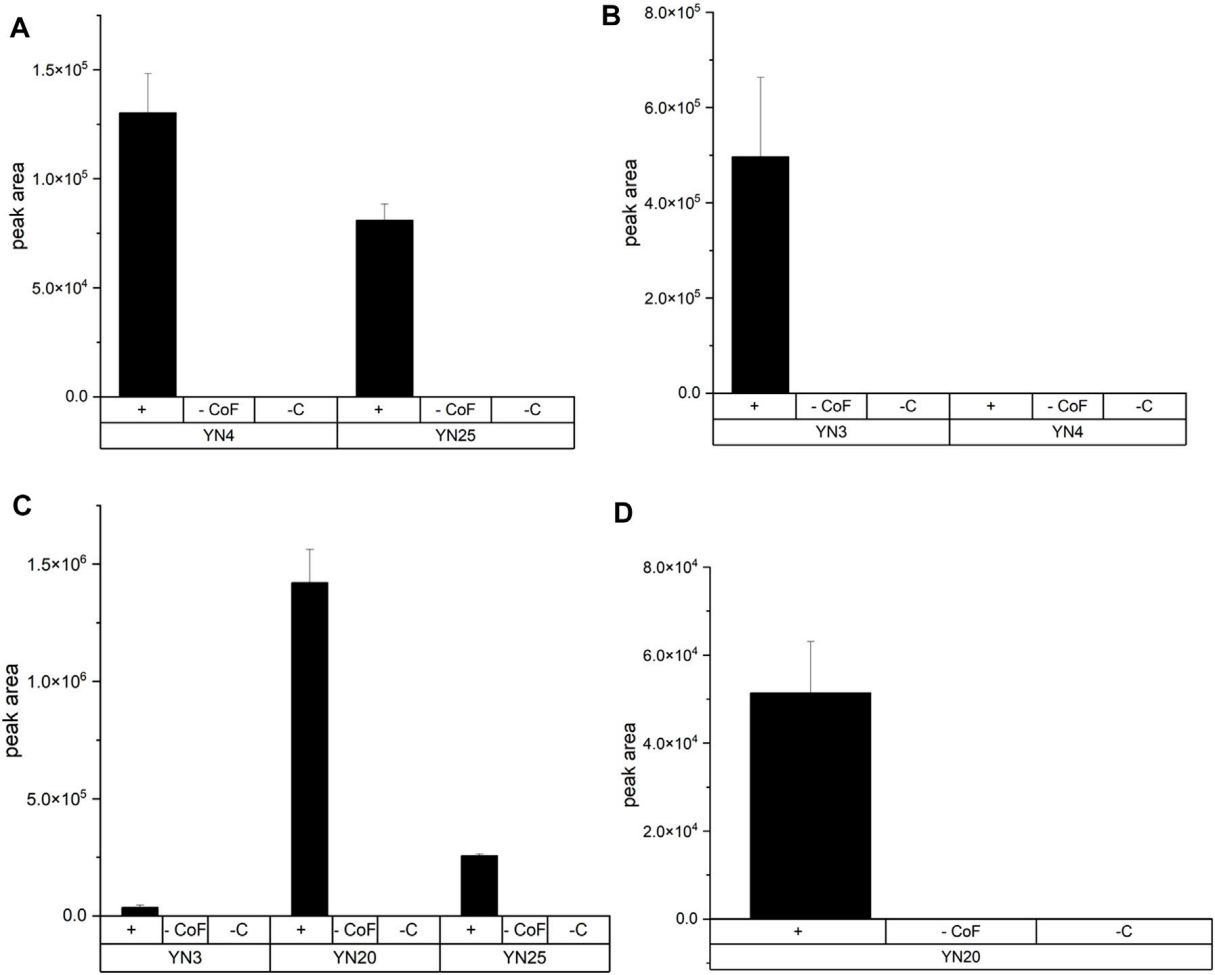
Production of three different sulfoconjugated metabolites by various human SULTs. Experimental conditions are described in the text. Names of fission yeast strain are given in brackets. +: incubation with cells and cofactors—CoF: incubation without cofactors but with cells—C: incubation without cells but with cofactors. **(A)** Production of DHEASU from DHEA. **(B)** Production of 7HCSU from 7HC. **(C)** Production of 4HPSU from 4HP. Each experiment was done in triplicates. **(D)** Production of SASU from SA.

As proof of concept the above-mentioned standard protocol developed with the SULT1A3 (YN20) and 4HP was then applied to 7HC, DHEA, salbutamol (SA) and 4HP using various SULTs (Figure 3). Experimental conditions were as follows: 11 mM ATP, 5.5 mM (NH_4_)_2_SO_4_, 100 µM substrate, 20 mM MgCl_2_, and 2.5 $\times 10^{8}$ cells per sample in NH_4_HCO_3_ buffer (pH 7.8). Substrates were incubated with SULTs reported in literature to metabolize the respective substrates (Miyano et al., 2005; Gamage et al., 2006; Ko et al., 2012; Nishikawa et al., 2018). DHEA was transformed to dehydroepiandrosterone sulfate (DHEASU) by SULT1E1 (YN25) and SULT2A1 (YN4). The strain with the latter enzyme provided a higher space-time yield. In case of the substrates SA, 7HC, and 4HP with various enzyme isoforms phenolic sulfonated metabolites were found in all experiments except in incubations of 7HC with SULT2A1 (YN4). The generation of 4HPSU was catalysed by SULT1B1 (YN3), SULT1E1 (YN25), and SULT1A3 (YN20). In same order yields were ascending. SA sulfonation to salbutamol sulfate (SASU) was successfully performed by SULT1A3 (YN20). 7HCSU was generated by SULT1B1 (YN3). Blank incubations served as negative controls. All blank incubations either without cofactors (-CoF) or without cells (-C) did not result in any detection of sulfonated metabolites.

**FIGURE 4:**
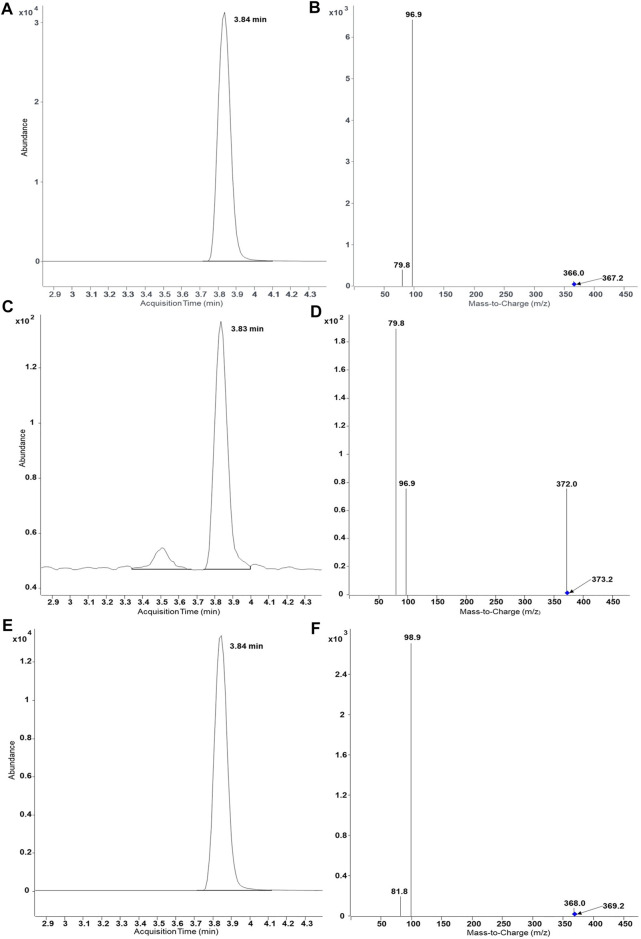
UHPLC-MS/MS results of biosynthesis of DHEASU, D_6_-DHEASU, and DHEA-^34^S-SU by SULT2A1 (YN4). Product ion spectra generated in ESI^−^ are dominated by the fragment HSO_4_^−^ (96.9) or H^34^SO_4_^−^ (98.9). **(A)** Chromatogram of DHEASU displaying qualifier transition *m/z* 367.2 → 96.9; **(B)** MRM transitions in DHEASU assay (*m/z* 367.2→366, 367.2→96.9 and 367.2→79.8); **(C)** Chromatogram of D_6_-DHEASU displaying qualifier transition *m/z* 373.2 → 96.9 **(D)** MRM transitions in D_6_-DHEASU assay (*m/z* 373.2→372, 373.2→96.9 and 373.2→79.8); **(E)** Chromatogram of DHEA-^34^S-SU displaying qualifier transition *m/z* 369.2 → 98.9; **(F)** MRM transitions in DHEA^34^SU assay (*m/z* 369.2→368, 369.2→98.9 and 369.2→81.8).

**FIGURE 5:**
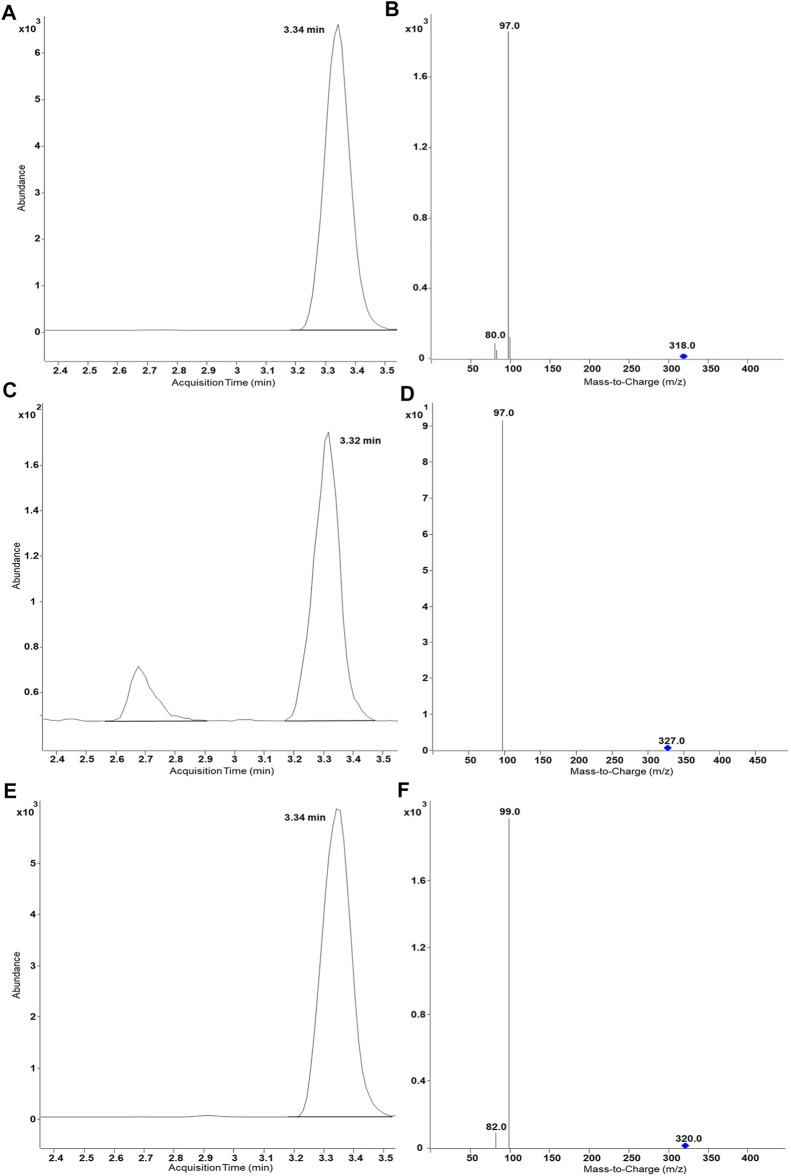
UHPLC-MS/MS results of biosynthesis of SASU, D_9_-SASU, and SA-^34^S-SU by SULT1A3 (YN20). Product ion spectra generated in ESI^−^ are dominated by the fragment HSO_4_^−^ (96.9) or H^34^SO_4_^−^ (98.9). **(A)** Chromatogram of SASU shows ion transition *m/z* 318.0 → 97; **(B)** MRM transitions in SASU assay (*m/z* 318.0→97.0 and 318.0→80.0); **(C)** Chromatogram of D_9_-SASU shows *m/z* 327.0 → 97.0; **(D)** MRM transitions in D_9_-SASU assay (*m/z* 327.0→97.0 and 327.0→79.8); **(E)** Chromatogram of SA-^34^S-SU shows *m/z* 320.0 → 99.0; **(F)** MRM transitions in SASU assay (*m/z* 320.0→99.0 and 320.0→82.0).

In the past, recombinant fission yeast strains that express human UDP glucuronosyltransferases were successfully used for the production of stable isotope-labelled glucuronides (Dragan et al., 2010; Buchheit et al., 2011). In order to demonstrate the usefulness of our new protocol for the production of stable isotope-labelled sulfometabolites, biotransformations with D_6_-DHEA, D_9_-salbutamol (D_9_-SA), and (NH_4_)_2_^34^SO_4_ were conducted in the present study (Figure 4 and Figure 5). Non-labelled DHEA or D_6_-DHEA were subjected to SULT2A1-dependend enzyme bag biotransformations either with (NH_4_)_2_SO_4_ or (NH_4_)_2_^34^SO_4_. Results of UHPLC-MS/MS analysis proved same retention time of DHEASU, D_6_-DHEASU, and DHEA-^34^S-SU (Figures 4A,C,E) the respective pattern of mass transitions (Figures 4B,D,F) showed the successful production of non-labelled and isotope-labelled sulfometabolites. The same strategy was performed using non-labelled SA and D_9_-SA with SULT1A3 (YN20) as well (Figure 5).

### Further Optimization of Buffer, (NH_4_)_2_SO_4_ and Substrate Concentrations

Unexpectedly, the sulfonation of 7HC by SULT2A1 (YN4) could not be shown using above mentioned conditions, even though the enzyme is known to metabolize this substrate (Nishikawa et al., 2018; Sun et al., 2020). It was suspected that product lability might be a reason. In order to confirm this suspicion, degradation assays were performed. Indeed, the incubation of 7HCSU with enzyme bags generated using YN3 at pH 7.8 led to more than 99% loss of the compound within 5 hours. By contrast, degradation tests in buffer without cells proved good stability of 7HCSU (Supplementary Figure S2). Apparently, there are endogenous fission yeast enzymes which can catalyze a cleavage of this sulfated metabolite.

**FIGURE 6:**
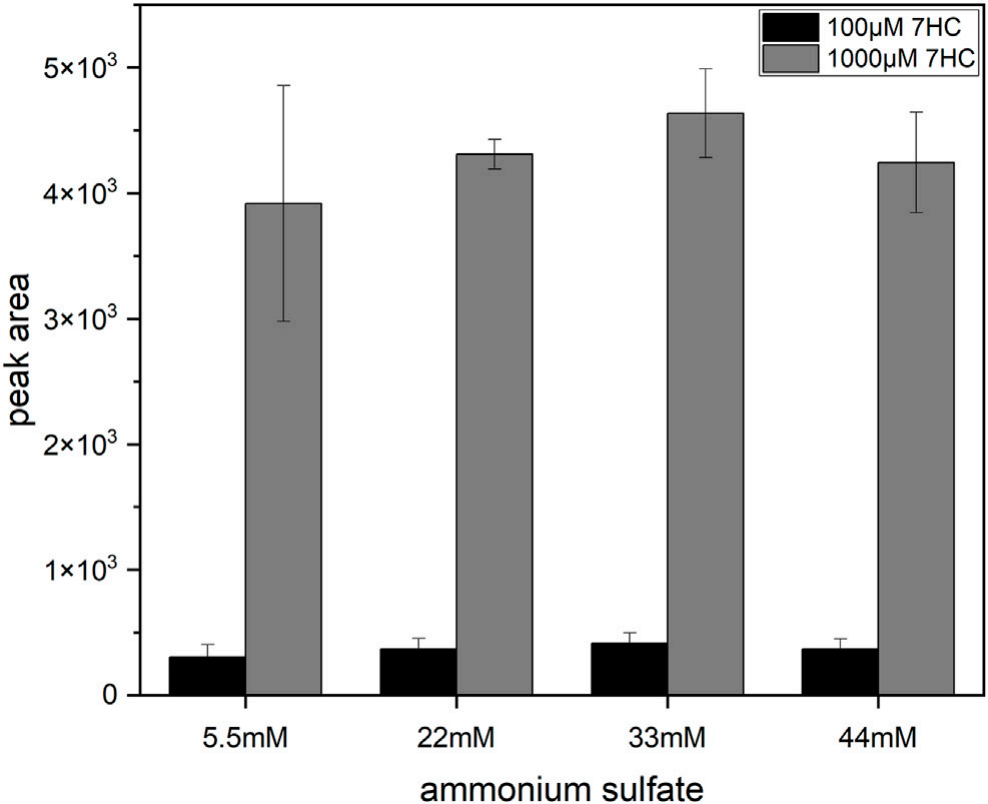
Influence of (NH_4_)_2_SO_4_ and substrate concentrations on 7HCSU formation from 7HC by SULT2A1. Each experiment was done in triplicates. Error bars show the standard deviations.

Further investigations of the degradation of 7HCSU were performed by incubating permeabilized YN3 cells either in NH_4_HCO_3_ buffer (at pH 7.8 or 7.4) or in phosphate buffer (at pH 7.8, 7.4, or 6.5). The results showed that in pH 7.8 phosphate buffer 7HCSU displays the smallest amount of degradation (Supplementary Figure S3). With the intention of avoiding total degradation of 7HCSU and increasing sulfonation yield of 7HC by SULT2A1 (YN4), and also with the purpose for exploring the possibility of further optimization of the general protocol, enzyme bag assays were subsequently conducted at higher substrate concentrations (7HC at 100 µM or 1 mM), higher (NH_4_)_2_SO_4_ concentrations (5.5, 22, 33, or 44 mM), and also in phosphate buffer (pH 7.8). The results demonstrated that the peak area of 7HCSU reached the highest levels at 1 mM substrate concentration, while the influence of the (NH_4_)_2_SO_4_ concentration on the yield was minor (Figure 6). The same optimization with higher substrate and ammonium sulfate concentrations was performed for the biotransformation of 4HP with SULT1A3 (YN20) as well. In this case, no particularly obvious differences were found among the different conditions (Supplementary Figure S4).

### Final Protocol

**FIGURE 7:**
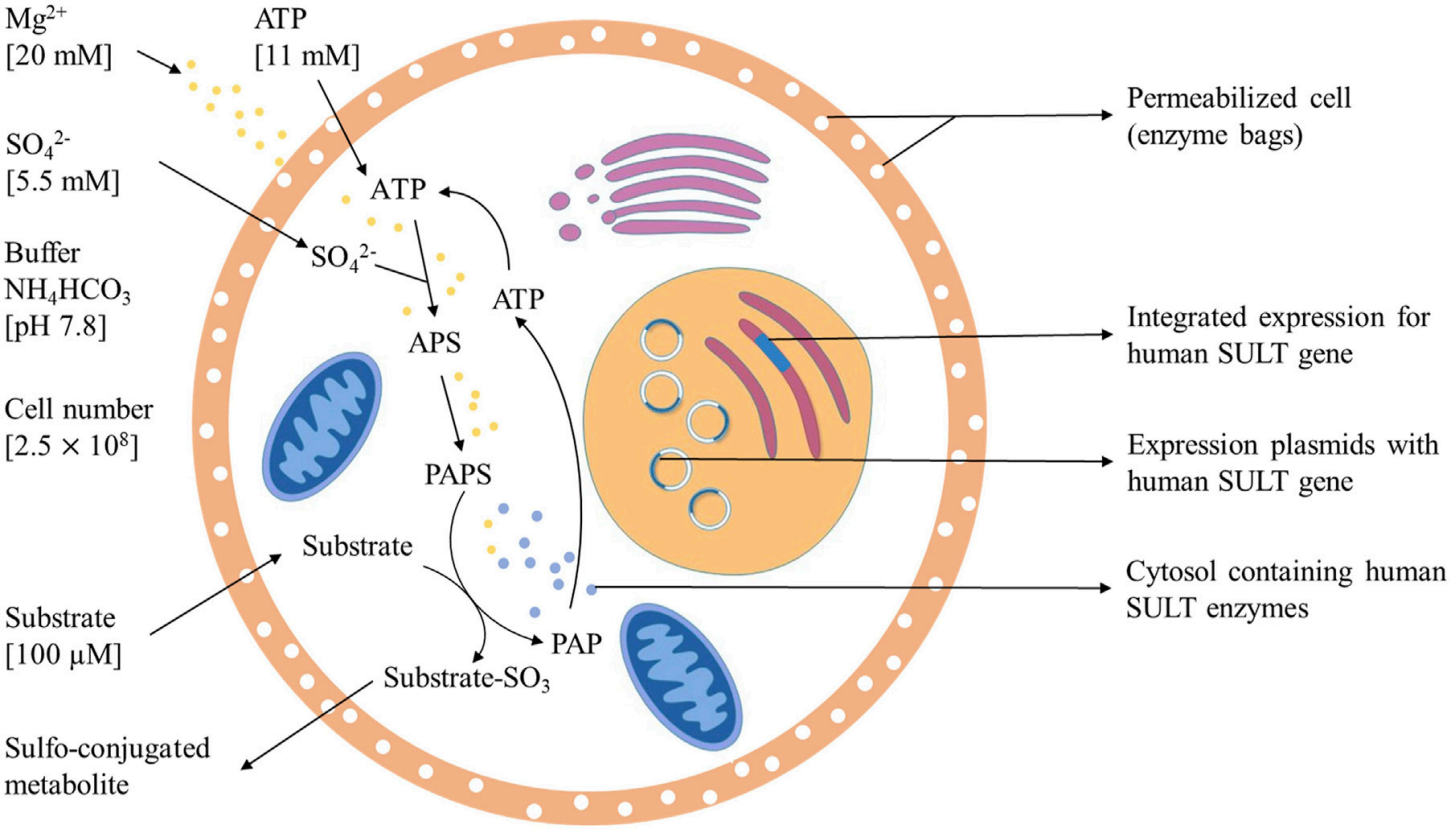
Optimized method for sulfotansferase catalyzed metabolite generation including PAPS replacement protocol.

The final protocol (Figure 7) for this sulfonation assay uses 20 mM Mg^2+^, 11 mM ATP, 5.5 mM SO_4_^2-^, and 2.5 × 10^8^ cells per incubation in ammonium bicarbonate buffer at pH 7.8 for 5 h at 37°C. In the case of 4HP, the most efficient substrate concentration was 100 µm. Sulfonation of compounds with a low affinity to a SULT can be achieved by enhancing substrate concentration to 1 mM.

## Discussion

In this study, the successful replacement of the SULT cofactor PAPS by ATP and (NH_4_)_2_SO_4_ in a recombinant sulfoconjugate biosynthesis system is reported. While production and regeneration of PAPS were previously described in a chemoenzymatic approach (An et al., 2017), in an enzymatic approach (Burkart et al., 2000), and in liver S9 fraction-based biosynthesis (Weththasinghe et al., 2018), this assay successfully combines both, PAPS (re-)generation and sulfonation of xenobiotics. Multifactorial optimization was performed applying DoE principles using the model substrate 4HP and human SULT1A3, which is expressed by recombinant fission yeast strain YN20 (Figure 1). Compared with the biosynthesis where PAPS was used as cofactor, the optimized method with ATP and (NH_4_)_2_SO_4_ resulted in a more than doubled yield of product (Figure 2). At the same time, the cost per experiment was reduced by a factor of 60. Further optimization was performed using 4HP with SULT1A3 and 7HC with SULT2A1. Permeabilized fission yeast (enzyme bags) assays with PAPS regeneration combine the advantage of high sensitivity and low cost. Small molecules like substrates, cofactors, and products can move in and out of the cells freely. Meanwhile, the enzymes needed for sulfonation remain trapped within the enzyme bags and can therefore be employed to catalyze the reactions of interest.

The concentration of magnesium ions was observed to be one of the most crucial parameters in the optimization process. Magnesium is an essential electrolyte in the human body. As a cofactor, magnesium participates in more than 300 enzyme systems that modulate multiple biochemical reactions in the body (Gröber et al., 2015a). In biological systems that generate PAPS or sulfonated metabolites, magnesium functions as an assistant inorganic ion (Burkart et al., 2000; Weththasinghe et al., 2018). In this study, the concentration of MgCl_2_ was tested in a range from 1 to 100 mM. Ultimately, 20 mM was found to be the optimal concentration. The results demonstrated that within certain limits, the MgCl_2_ concentration had an evident impact on the yield of 4HPSU. Although the mechanism of magnesium in biological sulfonation is not yet completely understood, magnesium is reported to be essential for the pathway of PAPS synthesis in *S. cerevisiae* (Thomas and Surdin-Kerjan, 1997). It is reasonable to assume that magnesium either functions as an enzyme activator or is involved in ATP production within the sulfonation system (Swaminathan, 2003; Gröber et al., 2015b).

By screening several ATP concentrations, it was observed that higher ATP concentrations (≥50 mM) led to a significant decline of yield in sulfonated product formation. As a structural analogue of PAPS, ATP has been reported to competitively inhibit the sulfonation of human M and P phenol sulfotransferase (SULT1A3, SULT1A1) (Rens-Domiano and Roth, 1987; Dooley et al., 1994). This property might be responsible for the effects observed in here as well.

Afterwards, the standard protocol was applied to additional substrates and SULTs (Figure 3). Furthermore, production of stable isotope-labelled sulfometabolites, with D_6_-DHEA, D_9_-salbutamol (SA), and (NH_4_)_2_^34^SO_4_ were achieved applying the established protocol as well (Figures 4, 5). The competence of SULT2A1 to sulfonate 7HC was reported by Nishikawa (Nishikawa et al., 2018) and Sun (Sun et al., 2020). However, using our PAPS replacing protocol, the enzymatic activity of SULT2A1 towards 7HC could not be demonstrated under initial standard protocol conditions. A possible reason is a rapid degradation of the product 7HCSU, presumably by cleavage of the sulfate group. Therefore, the degradation of 7HCSU was subsequently investigated in reaction mixtures with and without cells (Supplementary Figure S1). Degradation was only found in assays with cells, which indicated that the degradation of 7HCSU is a result of enzymatical catalysis rather than of chemical instability. Less degradation was observed when phosphate buffer (pH 7.8) was used instead of hydrogen carbonate buffer, which is in line with earlier reports of sulfatase inhibition by phosphate (Lee and Van Etten, 1975; Bostick et al., 1978; Metcalfe et al., 1979). Furthermore, using phosphate buffer higher product yields were obtained with both SULT2A1 and SULT1B1. Therefore, for 7HC sulfonation experiments with enzyme bags, the usage of phosphate buffer (pH 7.8) is superior to that of NH_4_HCO_3_ buffer (pH 7.8).

In a previous study by Burkart et al. (2000) the generation of PAPS from ATP and inorganic sulfate was also achieved using genetically modified *E. coli*. Highest yields were obtained when the sulfate concentration dramatically exceeded that of ATP. Consequently, we increased the (NH_4_)_2_SO_4_ concentration to 44 mM. However, the yield of 4HPSU did not show a significant rise with increasing (NH_4_)_2_SO_4_ concentrations. This might indicate that the concentration of PAPS is not the main limiting factor of 4HPSU yield in this case.

Being a known hydroxysteroid converting SULT, SULT2A1 shows low affinity to phenolic compounds like 7HC (Tibbs et al., 2015), which might explain the lack of sulfoconjugated metabolite in initial experiments. Therefore, the concentration of 7HC was increased to 1 mM to facilitate the enzymatic reaction. A remarkable increase of 7HCSU production (Figure 6) was observed. In this manner, the standard protocol was modified for biotransformation of low affinity substrates in enzyme bags.

The developed assay allows to determine whether a substrate is sulfonated by any one of the 14 human SULTs and also permits a comparison of their sulfonation activity. Due to the fact that the biosynthesized sulfoconjugates are not available as references, a quantitation of the results by UHPLC-MS/MS is not possible. Therefore, metabolite formation rates cannot be given in absolute values in this study.

The successful development of an optimized PAPS replacement protocol (details in Figure 7) provides an economic and efficient way for further research of SULT-depended phase II metabolism pathways of drugs. Bigger scale screening experiments will be performed to demonstrate broad applicability and to further evaluate the possibilities of this great sulfonation technique. The biotechnological generation of sulfonated compounds and metabolites may be achieved on reasonable costs applying this assay.

## Data Availability

The original contributions presented in the study are included in the article/Supplementary Material, further inquiries can be directed to the corresponding authors.
