# Supplementary material for: Human Sulfotransferase Assays With PAPS Production in situ
Source: Front Mol Biosci. 2022 Feb 28;9:827638. doi: 10.3389/fmolb.2022.827638 (PMC8914157; doi:10.3389/fmolb.2022.827638)
Supplement: Supplementary file 1 [file DataSheet1.PDF]

Supplementary Material

1 Supplementary tables

Supplementary table 1. Chromatographic separation conditions

LC Method 1

**Stationary phase**	phenyl-hexyl, superficially porous particles (2.1 × 100 mm, 2.7 µm)	
**Eluent A**	10 mM ammonium formate and 0.1 % formic acid in water	
**Eluent B**	10 mM ammonium formate and 0.1 % formic acid in acetonitrile/water (9/1 [v/v])	
**Gradient 1**	[4HP/4HPSU]	
	0 - 1 min	0 % B
	1 - 4 min	0-30 % B
	4 - 4.5 min	30-95 % B
	4.5 - 5.5 min	95 % B
	5.5 - 5.6 min	95-0 % B
**Flow rate**	0.3 ml/min	
**Temperature**	20 °C	
**Injection volume**	1 µl	

LC Methods 2 - 5

**Stationary phase**	C18, end capped, superficially porous particles (2.1 × 50 mm, 1.9 µm)			
**Eluent A**	10 mM ammonium formate and 0.1 % formic acid in water			
**Eluent B**	10 mM ammonium formate and 0.1 % formic acid in acetonitrile/water (9/1 [v/v])			
**Injection volume**	1 µl			
**Gradients 2 & 3**	[4HP/4HPSU]		[7HC/7HCSU]	
	2 min	0-10 % B	3 min	0-20 % B
	3.5 min	10-95 % B	2.5 min	20-95 % B
	0.4 min	95 % B	0.4 min	95 % B
	0.1 min	95-0 % B	0.1 min	95-0 % B
**Flow rate**	0.3 ml/min		0.3 ml/min	
**Temperature**	20 °C		35 °C	
**Gradients 4 & 5**	[DHEA/DHEASU]		[SA/SASU]	
	3 min	0-50 % B	3 min	0-5 % B
	3 min	50-95 % B	2.9 min	5-95 % B
	1 min	95 % B	0.1 min	95-0 % B
	0.1 min	95-0 % B		
**Flow rate**	0.3 ml/min		0.3 ml/min	
**Temperature**	35 °C		35 °C	

Supplementary Table 2: MS/MS operating parameters.

**Mass Detector**	**Triple Quadrupole MS**			
**Ion Source**	AJS ESI^+			
**Scan Type**	MRM			
**Source parameter ESI^+ / ESI^-**				
	4HP/<br>4HPSU	7HC/<br>7HCSU	DHEA/<br>DHEASU	SA/<br>SASU
**Gas temperature**	160 °C	290 °C	230 °C	240 °C
**Drying Gas**	11 L/min	20 L/min	20 L/min	20 L/min
**Nebulizer**	30 psi	25 psi	25 psi	25 psi
**Sheath gas heater**	375 °C	400 °C	400 °C	400 °C
**Sheath gas flow**	12 L/min	12 L/min	10 L/min	12 L/min
**Capillary**	3000 V	3000 V / 4500 V	3000 V / 4500 V	4000 V / 3000 V
**Charging**	1500 V	500 V / 0 V	500 V / 0 V	500 V / 1500 V

Supplementary Table 3: Transitions for all analytes.

Analyte	Precursor Ions (m/z)	Product Ions (m/z)	CE (V)	ESI
**4HPSU**	356	276	20	+
	356	116	20	+
	354	158	25	-
	354	80	25	-
**4HP**	276.2	116.1	16	+
	276.2	72	12	+
	276.2	58	44	+
**7HCSU**	243	162.9	16	+
	243	107	32	+
	243	91	40	+
	243	77	56	+
**7HC**	163.04	119.1	20	+
	163.04	107	24	+
	163.04	91	24	+
	163.04	77.1	36	+
	163.04	51.1	56	+
**DHEA**	289.2	271.1	4	+
	289.2	253.1	8	+
	271.2	253.1	12	+
	271.2	213.2	12	+

Supplementary Table 3 (cont.): Transitions for all analytes.

**Analyte**	**Precursor Ions (m/z)**	**Product Ions (m/z)**	**CE (V)**	**ESI**
**D_6-DHEA**	295.2	277.1	4	+
	295.2	259.1	8	+
**DHEASU**	367.2	366	12	-
	367.2	96.9	44	-
	367.2	79.8	60	-
**D_6-DHEASU**	373.2	372	12	-
	373.2	96.9	44	-
	373.2	79.8	60	-
**DHEA-^{34}S-SU**	369.16	368	12	-
	369.16	98.9	44	-
	369.16	81.8	60	-
**SA**	240	222	8	+
	240	166	12	+
	240	147.9	16	+
	240	91	48	+
	240	77.1	56	+
**D_9-SA**	249.2	231.1	8	+
	249.2	148.6	20	+
	249.2	121	36	+
	249.2	77	64	+
	249.2	66	32	+

Supplementary Table 3 (cont.): Transitions for all analytes.

Analyte	Precursor Ions (m/z)	Product Ions (m/z)	CE (V)	ESI
**SASU**	320	222	20	+
	320	166	20	+
	320	57	32	+
	318	97	25	-
	318	80	25	-
**D_9-SASU**	329	231.1	8	+
	329	166	20	+
	329	148.6	20	+
	329	66	32	+
	327	97	8	-
**SA-^{34}S-SU**	322	222	20	+
	322	166	20	+
	322	57	32	+
	320	99	25	-
	320	82	25	-

2 Supplementary Figures

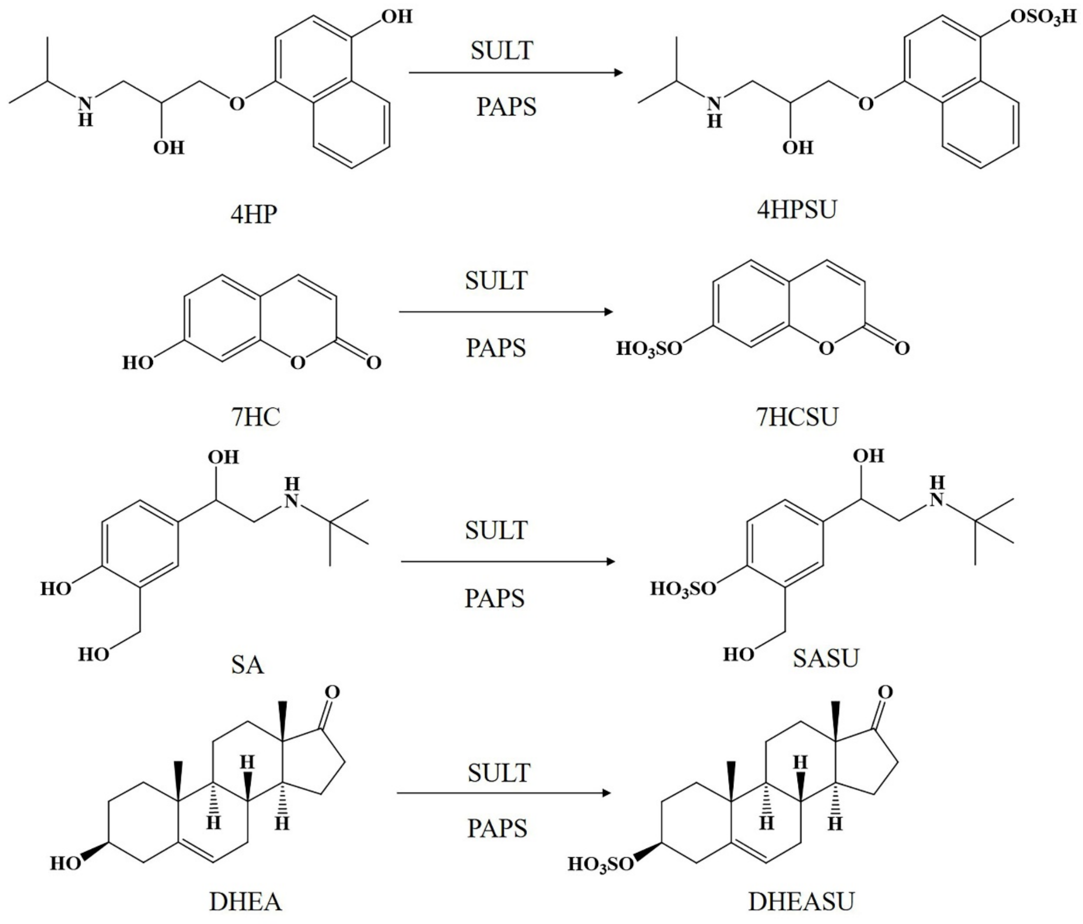

Supplementary Figure 1. Reaction schemes.

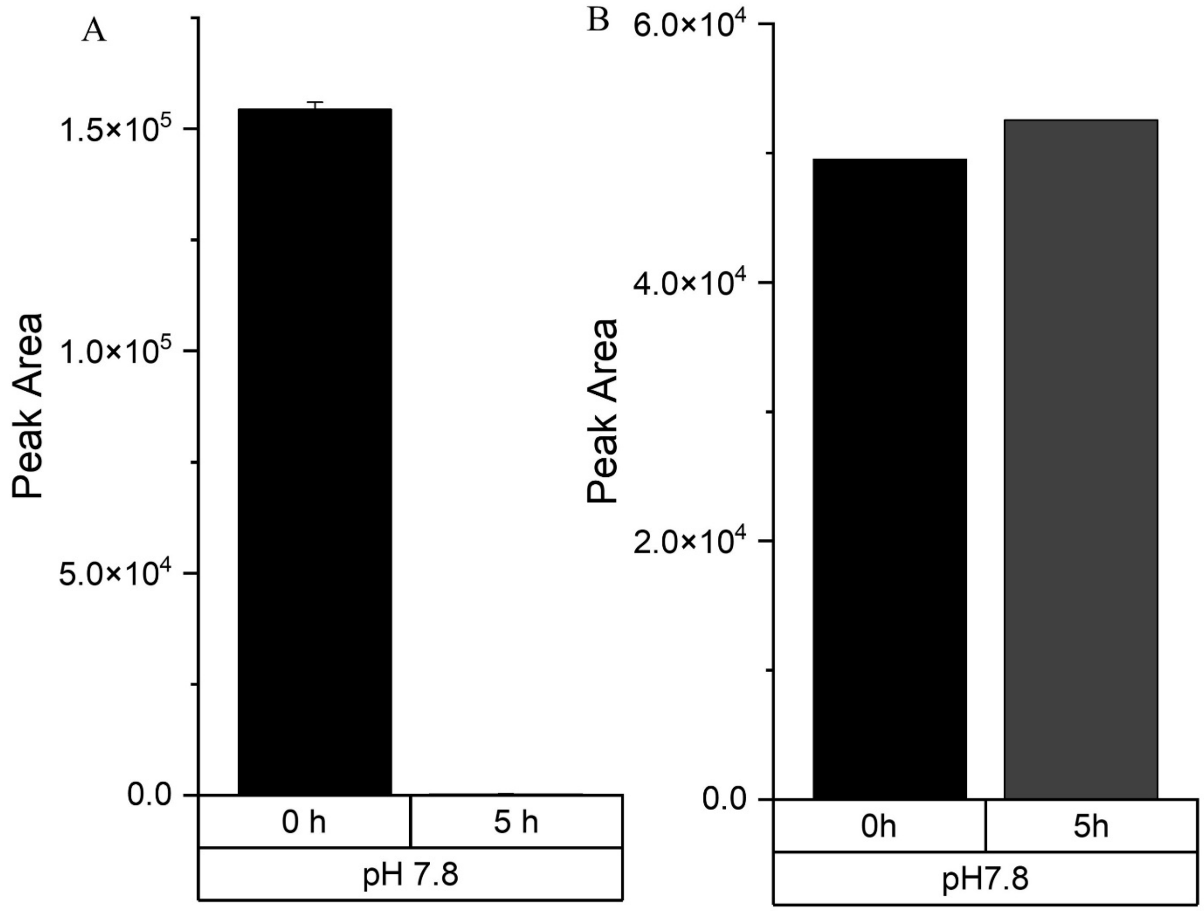

Supplementary Figure 2. Degradation of 7HCSU under different incubation conditions. (A) Incubation of 7HCSU with enzyme bags generated from strain YN3 in NH_4HCO_3 buffer (pH7.8); (B) Incubation of 7HCSU in NH_4HCO_3 buffer (pH7.8) without cells.

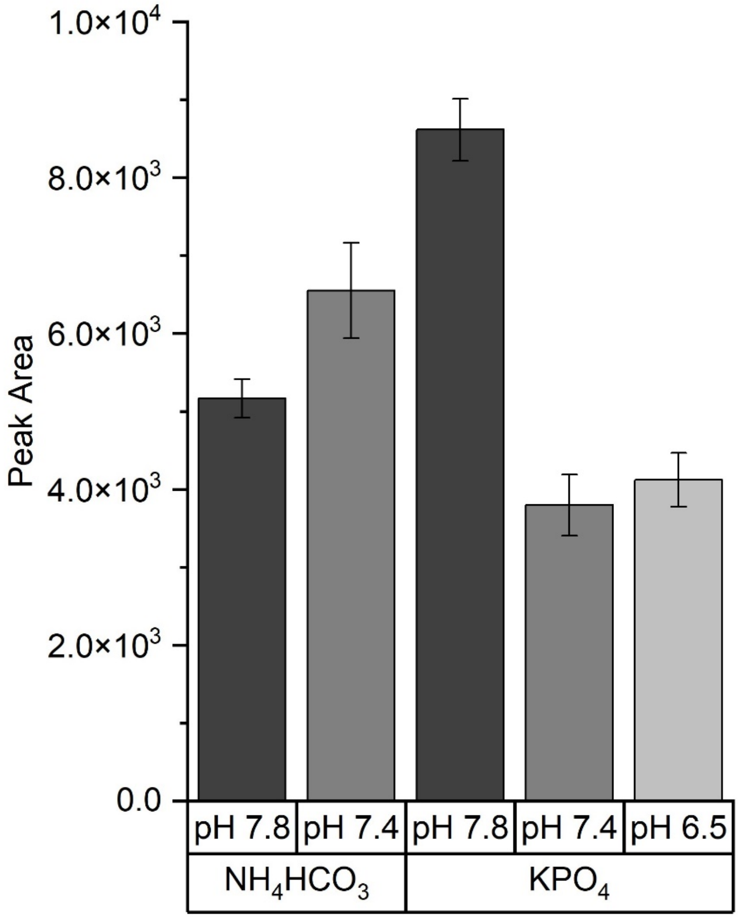

Supplementary Figure 3. Comparison of the SULT1B1-dependend production of 7HCSU from 7HC under different buffer conditions.

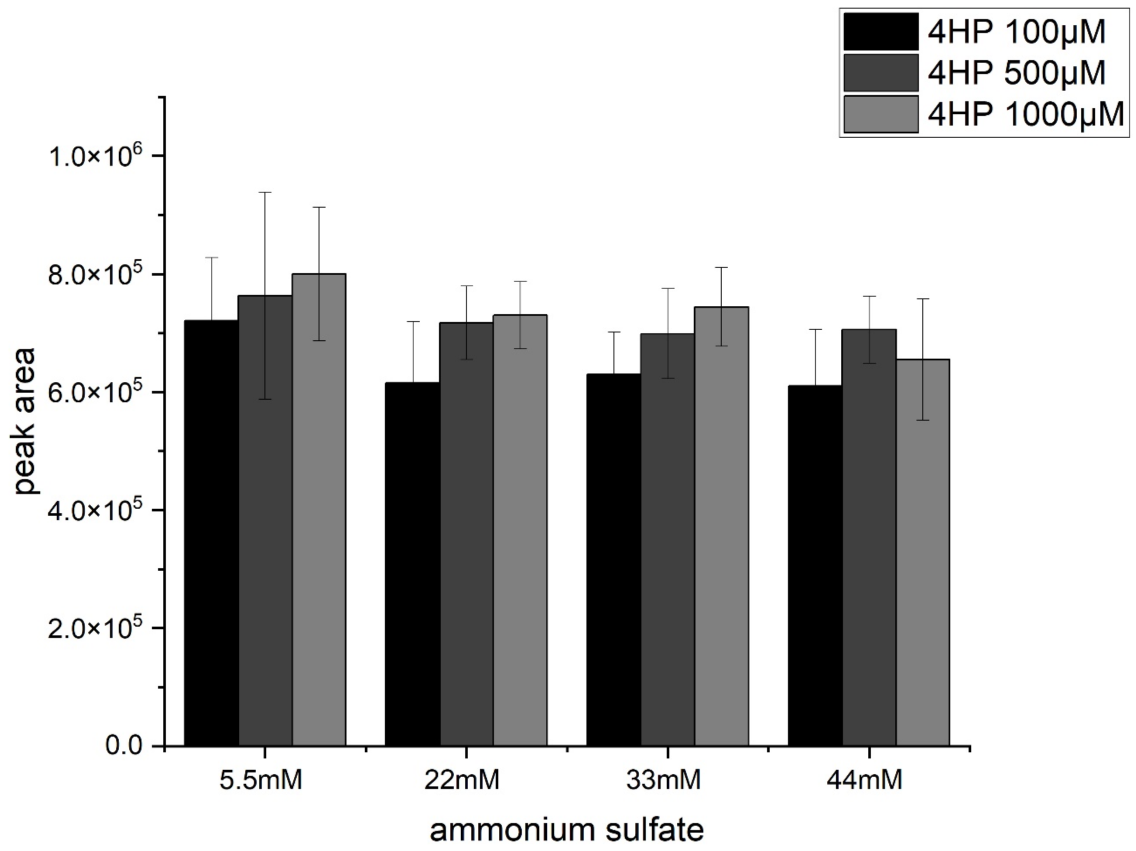

Supplementary Figure 4. Influence of ammonium sulfate and 4HP concentration on 4HPSUformation. 4HPSU produced by 4HP incubated with SULT1A3 under different conditions. Each experiment was conducted in triplicates.
